# Ga_2_O_3_(Sn) Oxides for High-Temperature Gas Sensors

**DOI:** 10.3390/nano11112938

**Published:** 2021-11-02

**Authors:** Nataliya Vorobyeva, Marina Rumyantseva, Vadim Platonov, Darya Filatova, Artem Chizhov, Artem Marikutsa, Ivan Bozhev, Alexander Gaskov

**Affiliations:** 1 Chemistry Department, Moscow State University, 119991 Moscow, Russia; natali.vorobyeva@gmail.com (N.V.); agnes1992@yandex.ru (V.P.); gak1.analyt@gmail.com (D.F.); chizhov@inorg.chem.msu.ru (A.C.); artem.marikutsa@gmail.com (A.M.); gaskov@inorg.chem.msu.ru (A.G.); 2 Quantum Technology Center, Moscow State University, 119991 Moscow, Russia; bozhjev.ivan@physics.msu.ru; 3 Faculty of Physics, Moscow State University, 119991 Moscow, Russia

**Keywords:** oxide materials, semiconductor gas sensor, Ga_2_O_3_, doping, carbon monoxide CO, ammonia NH_3_

## Abstract

Gallium(III) oxide is a promising functional wide-gap semiconductor for high temperature gas sensors of the resistive type. Doping of Ga_2_O_3_ with tin improves material conductivity and leads to the complicated influence on phase content, microstructure, adsorption sites, donor centers and, as a result, gas sensor properties. In this work, Ga_2_O_3_ and Ga_2_O_3_(Sn) samples with tin content of 0–13 at.% prepared by aqueous co-precipitation method were investigated by X-ray diffraction, nitrogen adsorption isotherms, X-ray photoelectron spectroscopy, infrared spectroscopy and probe molecule techniques. The introduction of tin leads to a decrease in the average crystallite size, increase in the temperature of β-Ga_2_O_3_ formation. The sensor responses of all Ga_2_O_3_(Sn) samples to CO and NH_3_ have non-monotonous character depending on Sn content due to the following factors: the formation of donor centers and the change of free electron concentration, increase in reactive chemisorbed oxygen ions concentration, formation of metastable Ga_2_O_3_ phases and segregation of SnO_2_ on the surface of Ga_2_O_3_(Sn) grains.

## 1. Introduction

The materials based on Ga_2_O_3_ have attracted great attention for electronic applications because of its exceptional properties, such as large bandgap, high transmittance in the deep ultraviolet region, physical and chemical stability [1,2]. Of particular interest is the possibility to create Ga_2_O_3_-based materials for highly sensitive and selective resistive gas sensors [1,3,4], which can operate at high-temperatures (≥500 °C), for real-time monitoring of automobile exhaust gases and flue pollutant gases. The problem is that the most common sensors (based on semiconductor oxides SnO_2_, ZnO, WO_3_) for detecting CO, NH_3_ and hydrocarbons work at low or medium temperatures (250–350 °C) and an increase in the measurement temperature over 500 °C leads to a significant decrease in the sensor response. Moreover, strong reducing gases (such as CO), especially in an oxygen-deficient atmosphere at high temperatures, can react with oxide’s lattice oxygen, which can lead to a decrease in the stability and restoring of resistive type sensors [5]. Ga_2_O_3_-based materials are perspective for the detection of both oxidizing (O_2_ [3,6,7]) and reducing gases such as CO [7,8], H_2_ [3,7], CH_4_ [3], ethanol [3,9] and acetone [9] in high temperature environment. However, pristine Ga_2_O_3_ has a too low conductivity that limits its widespread use. Doping of Ga_2_O_3_ with consciously chosen metal ions provides an opportunity to regulate the electrical properties over a wide range. However, at the same time, the introduction of any extrinsic donor leads to a complex distribution of these elements between surface and volume of grains of semiconductor oxide matrix that affects its microstructure and surface reactivity and alters the gas sensor properties. Introduction of Sn(IV) in Ga_2_O_3_ structure can dramatically improve the conductivity of the material while retaining the high chemical and thermal stability of β-Ga_2_O_3_ for high-temperature gas sensor applications.

In this paper, we report on the synthesis of nanocrystalline materials Ga_2_O_3_ and Ga_2_O_3_(Sn) with different tin content, and the established correlations between their phase composition, microstructure parameters, surface reactivity and sensor properties when detecting carbon monoxide and ammonia. We propose materials suitable for high temperature gas sensors for real-time monitoring of automobile exhaust gases and flue pollutant gases from the energy conversion and chemical technologies processes, such as combustion of biofuels, organic waste, wood, etc. According to [10], after the second grade of the new German emission law for firewood-fueled low-power fireplaces came into effect in 2015, the upper emission CO limits for firewood-fueled single-room fireplaces is 1250 mg/m^3^ (1073 ppm), for central heaters is 400 mg/m^3^ (343 ppm) and are higher than the typical emissions from heating oil burners—about 50 mg/m^3^ CO (43 ppm). The maximum safe level of ammonia is 30 ppm according to WHO guidelines and 25 ppm according to the Occupational Safety and Health Administration (OSHA) [1]. Therefore, the sensor properties of synthesized materials were studied when detecting CO and NH_3_ in the concentration range from 12 to 260 ppm.

## 2. Materials and Methods

### 2.1. Materials Synthesis

Ga_2_O_3_(Sn) powders with different Sn content were synthesized by the co-precipitation method. The Ga(NO_3_)_3_·8H_2_O and SnCl_4_·5H_2_O were used as precursors, distilled water was added to form the solution with total metal ions concentration of 0.2 M. The concentration of Sn^4+^ was varied in the range of [Sn]/([Ga] + [Sn]) = 0–10 at.% (Table S1, Supplementary materials). Then, aqueous 12.5% ammonia solution was added dropwise to the stirred solution of metal salts up to the weakly alkaline pH. The white gel precipitate was formed according to the reaction scheme:

Ga^3+^ + 3 NH_3_·H_2_O → GaOOH↓ + 3 NH_4_^+^ + H_2_O, (1)

Sn^4+^ + 4 NH_3_·H_2_O + (α-2) H_2_O → SnO_2_·αH_2_O↓ + 4 NH_4_^+^. (2)

After aging for 30 min at room temperature, the precipitate was separated by centrifugation, washed 5 times with distilled water to remove residual ions and then dried at 50 °C for 24 h. Pure and tin-doped Ga_2_O_3_ powders were prepared by annealing the xerogels at different temperatures (500–1000 °C) for 24 h in air.

### 2.2. Materials Characterization

The process of the xerogel decomposition was carried out by the thermogravimetry (TG) and differential thermal analysis (DTA) on a simultaneous thermal analyzer STA 409 PC Luxx (Netzsch, Selb, Germany). The heating was effectuated in air flow (30 mL/min) in the temperature range of 40–1000 °C with a heating rate of 10 °C/min. Mass-spectrometric analysis of gaseous products was carried out using a quadrupole mass spectrometer QMS 403C Aёolos (Netzsch, Selb, Germany).

The elemental composition of xerogels was determined by inductively coupled plasma mass spectrometry (ICP-MS) on an Agilent 7500c quadrupole mass spectrometer (Agilent Technologies, Waldbronn, Germany). The measurements of analytical signals and data processing were performed using Agilent 7500 Series ICP-MS ChemStation (version G1834B, Agilent Technologies, Waldbronn, Germany) built-in software. For determination of tin and gallium, the following isotopes were used: ^118^Sn, ^120^Sn, ^69^Ga and ^71^Ga. The samples were weighed on a Sartorius 1702MP8 balance (Sartorius Lab Instruments GmbH, Goettingen, Germany) with ±0.1 mg precision. A LabMate 100–1000 μL dispenser (Warsaw, Poland), 1–5 mL and 2–10 mL dispensers (Thermo Scientific, Vantaa, Finland) and 15 mL polypropylene tubes (Greiner Bio_One GmbH, Frickenhausen, Germany) were used. To dissolve the samples, a mixture of HCl and HF 1:1 acids were added to 5–10 mg of the sample in a Teflon autoclave (CEM Corporation, Matthews, NC, USA). Then samples were digested in a closed type MARS-5 microwave reaction system with 12 XP_1500 Plus high-pressure vessels (CEM Corporation, Matthews, NC, USA) at the temperature of 220 °C within an hour. The working frequency of the system was 2455 MHz, the radiated power was 800 W. Throughout this paper, composition of samples obtained by ICP-MS will be given as the ratio *x* = [Sn]/([Ga] + [Sn]) × 100% (at.%).

Phase composition and crystal structure were determined by X-ray diffraction (XRD) using DRON-4-07 diffractometer (Bourevestnik JSC, St. Petersburg, Russia), Cu Kα radiation, λ = 1.5418 Å. The crystallite size (d_XRD_) of Ga_2_O_3_ was calculated from the broadening of the XRD peaks using the Scherrer equation.

The specific surface area of the samples was measured by low-temperature nitrogen adsorption on a Chemisorb 2750 instrument (Micromeritics, Norcross, GA, USA) using single point BET (Brunauer, Emmett, Teller) method. All samples were kept in a flow of helium (50 mL/min) for 1 h at 250 °C in a flow-through quartz reactor. To study nitrogen adsorption, a gas mixture of N_2_/He (30 vol.% N_2_) was passed through the system (12 mL/min); liquid nitrogen was used for cooling. The amount of adsorbed nitrogen was calculated from the change in the thermal conductivity of the gas mixture.

The morphology of the powders was studied by scanning electron microscopy (SEM) using a Carl Zeiss SUPRA 40 FE-SEM instrument (Carl Zeiss AG, Oberkochen, Germany) with Inlens SE detector (accelerating voltage 5 kV, aperture 30 μm).

The surface composition and chemical state of the elements were studied by X-ray photoelectron spectroscopy (XPS) on an Axis Ultra DLD spectrometer (Kratos Analytical Ltd., Manchester, UK). The spectra were obtained with monochromatic Al Kα radiation in an ultrahigh vacuum (10^−8^ Torr), emission current 10 mA. The charge shift was corrected using the peak of the C1s ground state with a binding energy of 285 eV. The survey spectra were recorded in the range of 1350–0 eV with a step of 0.5 eV. The spectra for the regions of the elements were obtained with a step of 0.1 eV. The obtained XPS spectra were fitted by mixed Gaussian-Lorentzian functions. 

The Fourier-transform infrared (FTIR) absorption spectra were recorded on a Frontier spectrometer (Perkin Elmer Inc., Beaconsfield, UK) in the 4000–400 cm^−1^ range with a resolution of 4 cm^−1^ and accumulation of 4 scans. The samples were pressed into KBr tablets. The in situ diffuse reflectance infrared Fourier transform (DRIFT) spectra were recorded on a Frontier spectrometer (Perkin Elmer) with a DiffusIR attachment and an HC900 (Pike Technologies, Madison, WI, USA) test chamber equipped with a heater and a ZnSe window. Spectra were recorded in the 4000–650 cm^−1^ range, accumulation of 30 scans, under laboratory conditions with automatic compensation for the level of H_2_O/CO_2_. Weighed portions of the samples (30 mg) were placed in an alundum crucible. The adsorption of probe molecules NH_3_ and CO_2_ on the surface of the samples was carried out at room temperature using the flow of a gas mixture containing 200–500 ppm NH_3_ and CO_2_ in air. The samples were preliminarily prepared at 150 °C in a flow of dry air for 1 h to remove adsorbed water, and then they were kept in the same atmosphere at room temperature for 1 h.

For the gas sensing experiments, the materials were mixed with a vehicle (α-terpineol in ethanol) and deposited in the form of thick films over functional substrates, provided with Pt contacts on the front side and a Pt-meander that acts both as heating element and temperature probe, on the back-side. Thick films were dried at 50 °C for 24 h and sintered at 500 °C for 5 h in air. All sensor measurements have been carried out by flow through technique under a controlled constant flux of 100 mL/min. The atmosphere composition was pre-assigned by means of electronic mass-flow controllers (Bronkhorst High-Tech B.V., Ruurlo, Netherlands), mixing flows coming from certified bottles containing a given amount of the target gas diluted in synthetic air with the background flow. The background atmosphere was obtained from a pure air generator. The certified gas mixtures (2560 ppm CO in N_2_; 2590 ppm NH_3_ in N_2_) and dry air (from pure air generator) were used as gas sources. Initially, the samples were kept for 1 h in air (flow 100 mL/min) at a temperature of 500 °C, then measurements were alternated in the presence of different concentrations of CO or NH_3_ (15 min) and in pure air (30 min). To investigate the reproducibility and stability of the sensor response, measurements were carried out several times. The sensor response *S* was calculated as

*S* = (*G_gas_* − *G_air_*)/*G_air_*, (3)

where *G_gas_*—conductance of the sample in the presence of reducing gas CO or NH_3_, *G_air_*—conductance in the pure air.

## 3. Results and Discussion

### 3.1. Precursor Characterization

The interaction of gallium and tin salts solution with aqueous ammonia leads to the formation of white precipitates. After drying at 50 °C the only crystal GaOOH phase was observed by XRD for all investigated samples (Figure S1, Supplementary Materials). The cation composition of the samples *x* = [Sn]/([Ga] + [Sn] (at.%) determined by ICP-MS varies from 0 to 13 at.% Sn and will be used further while discussing results in this paper. Table 1 represents the elemental and phase composition of synthesized Ga_2_O_3_ and Ga_2_O_3_(Sn) samples.

**Figure 1:**
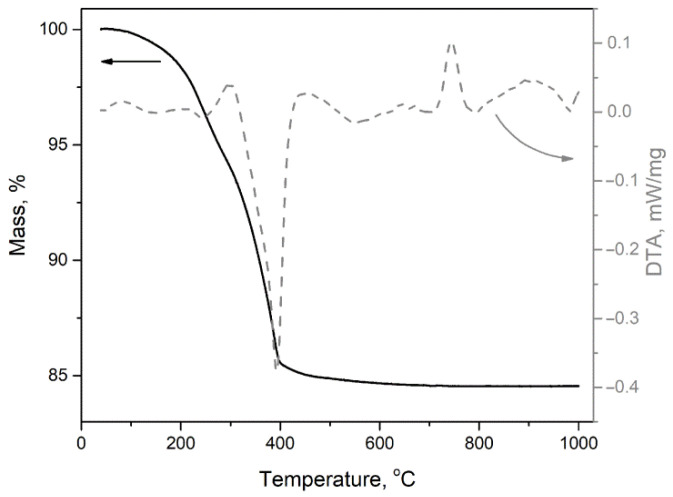
TG and DTA profiles of the GaOOH precursor.

The thermogravimetric analysis (TG), differential thermal analysis (DTA) and mass-spectrometry of gaseous products were performed to determine the conditions for the xerogel decomposition (Figure 1). The mass loss of the xerogel, which does not contain tin, was 15.5 %, while the theoretical mass loss when converting GaOOH to Ga_2_O_3_ is 9%. Thus, it can be assumed that the precipitate contains some amount of water or other phases, for example, gallium(III) hydroxide, which are most likely amorphous, since they are not detected by the XRD method. A strong endothermic peak on the DTA curve at about 390 °C indicates the decomposition of the precursor. Therefore, the minimum temperature of annealing, providing a complete precursor decomposition, was chosen as 500 °C for all samples.

An exothermic peak on the DTA curve at 740 °C is associated with a phase transition of α-Ga_2_O_3_ into β-Ga_2_O_3_. The exothermic character of this phase transition confirms the metastable nature of the α-phase. This peak is not observed for samples containing more than 4.3 at.% of tin.

The decomposition of xerogels occurs with the release of H_2_O (m/z(H_2_O^+^) = 18, m/z(OH^+^) = 17), NO_2_ (m/z(NO_2_^+^ = 46, m/z(NO^+^) = 30) and NH_3_ (m/z(NH^+^) = 15), as it can be seen from mass-spectra of gaseous decomposition products (Figure S2, Supplementary materials). In all cases the thermal decomposition is completed at the temperature of about 480 °C. Thus, the annealing temperature should be above 480 °C for the synthesis of Ga_2_O_3_-based oxide materials. In this work, the annealing of oxide materials for gas sensors was carried out at temperatures of 500, 750, and 1000 °C.

### 3.2. Phase Composition of Ga_2_O_3_(Sn)

#### 3.2.1. Annealing Temperature 500 °C

**Figure 2:**
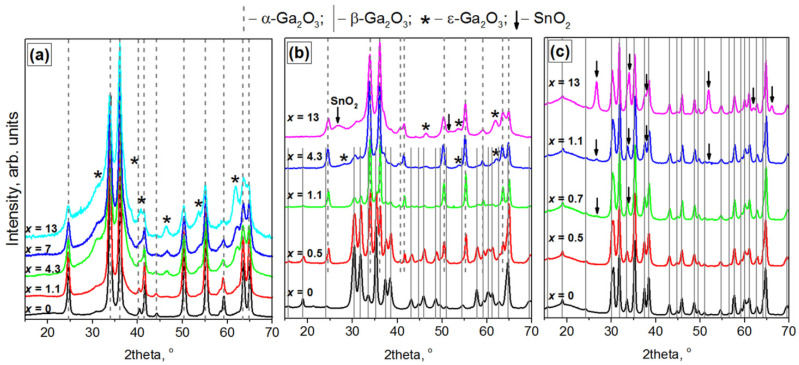
XRD patterns of Ga_2_O_3_(Sn), annealed at (**a**) 500, (**b**) 750 and (**c**) 1000 °C. Dash lines correspond to α-Ga_2_O_3_ phase; solid lines–to β-Ga_2_O_3_ phase; (*)—to ε-Ga_2_O_3_ phase; (↓)—to SnO_2_ phase.

The annealing of all GaOOH precursors containing 0–13 at.% Sn at 500 °C leads to the formation of a metastable α-Ga_2_O_3_ with a corundum structure as the main phase (Figure 2a, Table 1). The introduction of tin leads to a decrease in the average crystallite size from 18 ± 2 nm for x = 0 to 7 ± 1 nm for x = 13 at.% Sn, which is reflected by the broadening of the XRD peaks. The appearance of reflections of other phases is noticeable with the introduction of more than 4.3 at.% Sn. The unambiguous analysis of phase composition is difficult due to the superposition of the reflections of these phases with the peaks of main α-Ga_2_O_3_ phase and the broadening of all the peaks with the increasing of tin content. These secondary phases reflections can be assigned to the ε-Ga_2_O_3_ planes assuming a hexagonal lattice (see calculated XRD pattern in [11]) or the ε-Ga_2_O_3_ planes assuming an orthorhombic lattice (ICDD card (6–509), also described in [12]). In addition, the position of these peaks is in good agreement with cubic δ-Ga_2_O_3_ phase (ICDD card (6–529)), which was characterized as isomorphic to Tl_2_O_3_ or bixbyite (Mn,Fe)_2_O_3_ structures [12,13]. In more recent works it was shown that the δ-Ga_2_O_3_ does not exist as an independent phase and is a nanocrystalline form of the ε-Ga_2_O_3_ with an amorphous layer of nitrate and hydroxide groups around gallium oxide particles [2,11,14]. The formation of ε-Ga_2_O_3_ with increasing tin content will be discussed below for the samples obtained by annealing at 750 °C. The tin-containing phases are not clearly observed. However, at the location of the most intensive cassiterite reflections, one can see either broadening of the existing reflections of the gallium oxide phases or an increase in the background line for a sample containing 13 at.% Sn. Diffractograms of all samples are shown in Figures S3–S5 (Supplementary materials).

#### 3.2.2. Annealing Temperature 750 °C

XRD pattern of undoped gallium oxide obtained by annealing at 750 °C corresponds to pure thermodynamically stable β-Ga_2_O_3_ phase with a monoclinic structure (Figure 2b). This fact is in good agreement with both the numerous literature data indicating that at temperatures above 600–650°C the α-Ga_2_O_3_ → β-Ga_2_O_3_ transition occurs [2,11,13], and with our DTA data (an exothermic peak was observed on the DTA curve at 740 °C, corresponding to this phase transition, Figure 1).

For the samples with tin content up to 0.23 at.% the shape of the diffraction patterns remains practically unchanged, and the samples represent a thermodynamically stable β-Ga_2_O_3_ phase. The increase in tin concentration from 0.23 to 0.50 at.% leads to an increase in the content of α-Ga_2_O_3_ phase. The intensities of the two phases reflections are approximately the same. As tin concentration rises up to 0.7 at.% a sharp decrease in the content of the β-Ga_2_O_3_ phase occurs and the metastable α-Ga_2_O_3_ becomes the main phase. For the samples containing more than 7.0 at.% Sn β-Ga_2_O_3_ phase can be observed only by the broadening of α-Ga_2_O_3_ peaks or raising of a background line. It can be assumed that Sn(IV) cations “slow down” the phase α-Ga_2_O_3_ → β-Ga_2_O_3_ transition, shifting it to a higher temperature region. The absence of exothermic peaks in the DTA curves may indicate a very slow transition from one phase to another, which cannot be recorded under the measurement conditions used.

The samples with *x* > 0.5–0.7 at.% Sn also contain some other phases in addition to α- and β-Ga_2_O_3_ (Figure 2b). The reflections of these phases can be assigned to the ε-Ga_2_O_3_ planes [11,12,15]). The formation of ε-Ga_2_O_3_ phase in the presence of Sn(IV) cations may be explained by different factors [16]. First, with the increase in tin content an increase in the specific surface area occurs, and the surface energy begins to play an important role. The β-phase, which is thermodynamically more stable in the case of a bulk sample, may become metastable in comparison with the ε-phase stable in nanosized objects due to the high surface energy of this phase in comparison with other polymorphic modifications of gallium oxide [16]. However, ε-Ga_2_O_3_ phase is not always obtained for nanosized objects: in some cases, Ga_2_O_3_ with small crystalline sizes has only the β-phase structure [16]. Secondly, the formation of ε-Ga_2_O_3_ may be caused by the stabilization of this phase upon the introduction of tin into the crystal lattice of gallium oxide. The formation of ε-Ga_2_O_3_ was found during the tin-assisted growth of gallium oxide by plasma-assisted molecular beam epitaxy [15]. The possible mechanism of ε-Ga_2_O_3_ stabilization is related to the coordination environment of gallium and tin ions in their compounds. The ratio of tetrahedral and octahedral coordinated Ga^3+^ ions is 1:1 in β-Ga_2_O_3_. In ε-Ga_2_O_3_ number of the octahedral coordinated Ga^3+^ ions is higher than the tetrahedral ones. In the cassiterite SnO_2_ with rutile structure Sn^4+^ ions have an octahedral coordination with oxygen atoms. As a result, the introduction of Sn(IV) cations may favor the formation of the phase with more octahedral lattice sites. In addition, the bond lengths should be taken into consideration: the bond length in rutile SnO_2_ is longer than 2 Å, while for different Ga_2_O_3_ phases it is <1.9 Å for tetrahedral coordinated Ga^3+^ ions and >1.9 Å for octahedral coordinated ones [15]. The third factor that favors the formation of ε-phase is mechanical stress in the structure, which was observed for Ga_2_O_3_(Sn) films. Another possible explanation for the formation of ε-Ga_2_O_3_ phase can be the influence of residual chloride ions from SnCl_4_·5H_2_O, which was taken as the starting material. Thus, the authors of [17] associate the formation of the ε-phase with the presence of chlorine during the preparation of epitaxial α-, β-, and ε-Ga_2_O_3_ films by the MOCVD (metal-organic chemical vapor deposition) and HVPE (halide vapor phase epitaxy) methods. Chlorine can be incorporated into the structure to reduce the mechanical stresses. The ε-Ga_2_O_3_ phase has large distances in the crystal lattice compared to the α-phase, and therefore should more easily include chlorine impurities [17].

As for tin-containing phases, the presence of cassiterite is not clearly seen even for the sample containing 7 at.% Sn because of broadening of Ga_2_O_3_ reflections, however the increase in baseline at the SnO_2_ peaks positions is observed. The XRD pattern of the sample with *x* = 13 at.% Sn contains a wide peak in the range of 26–28 (Figure 2b), which is attributed to the SnO_2_ phase. The large width of this peak indicates the small size of SnO_2_ crystallites, which present possibly in the form of segregation on Sn-doped Ga_2_O_3_ grains.

The introduction of tin leads to the decrease in the average crystallite size of Ga_2_O_3_ phases as it was seen for annealing temperature of 500 °C. For samples with *x* = 0–1.1 at.% Sn the crystallite size of β-Ga_2_O_3_ phase is in the range of d_XRD_ = 12 ± 2 nm. With an increase in the tin content from *x* = 1.1 to *x* = 13 at.% Sn the d_XRD_ for α-Ga_2_O_3_ phase decreases from 22 ± 3 nm to 10 ± 2 nm. The decrease in the crystallite size with an increase in the tin content is consistent with the results obtained by low-temperature nitrogen adsorption method: the specific surface area increases from 11 ± 5 to 44 ± 5 m^2^/g with the increase in tin content from *x* = 0 to *x* = 13 at.% Sn.

#### 3.2.3. Annealing Temperature 1000 °C

According to X-ray diffraction data (Figure 2c), the samples obtained by annealing at 1000 °C and containing from 0 to 0.4 at.% Sn consist of the β-Ga_2_O_3_ phase. The average crystallite size is 20 ± 2 nm.

The small peak at 26.7 2θ is observed for the sample containing 0.7 at.% Sn that indicates the formation of tin dioxide with cassiterite structure. The increase in tin concentration leads to the growth of SnO_2_ phase content and less intensive SnO_2_ peaks can be observed. The average crystallite size of SnO_2_ phase is 13 ± 1 nm. The average crystallite size of β-Ga_2_O_3_ is 18 ± 2 nm.

The absence of gallium(III) oxide phases other than β-Ga_2_O_3_ is consistent with the literature data that only one thermodynamically stable β-Ga_2_O_3_ phase can be obtained by heating of all other polymorphic modifications in air [2,18].

### 3.3. Microstructure of Ga_2_O_3_(Sn)

**Figure 3:**
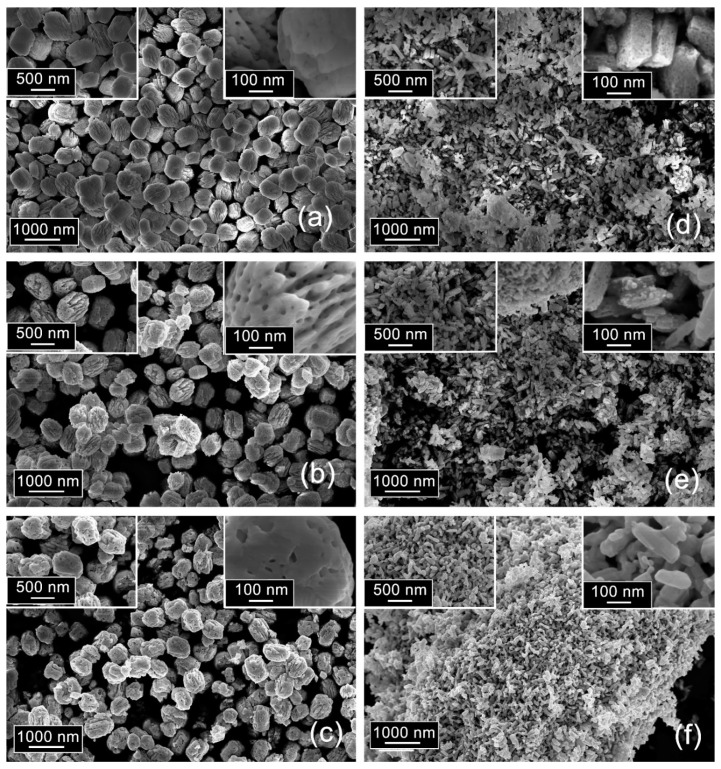
SEM images of Ga_2_O_3_ samples, annealed at (**a**) 500, (**b**) 750 and (**c**) 1000 °C; Ga_2_O_3_(Sn) samples (x = 13 at.%), annealed at (**d**) 500, (**e**) 750 and (**f**) 1000 °C.

SEM images of Ga_2_O_3_(Sn) (x = 0 and 13 at.%) are represented in Figure 3. Pure Ga_2_O_3_ (x = 0) consist of bud-like granules with the size of 400–600 nm. It can be seen that Ga_2_O_3_ particles are not the dense ones but appear as complex structures made of dozens of plate-like crystals. Pores with the diameter of 10–20 nm are observed within these plate-like particles.

The introduction of tin leads to a change in the shape of the agglomerates and a decrease in their size. The Ga_2_O_3_(Sn) sample (x = 13 at.%) annealed at 500 °C consists of elongated porous parallelepipeds with a hierarchical structure formed by plates. The length of such agglomerated particles is up to 400 nm, while the thickness lies in the range from 30 to 100 nm.

In both cases (x = 0 and 13 at.%), an increase in the annealing temperature does not lead to a change in the size of the agglomerates, but causes a change in the porous structure. An increase in the annealing temperature to 750 °C leads to the enlargement of the pores, and a further increase up to T = 1000 °C is accompanied by a significant decrease in their concentration.

**Figure 4:**
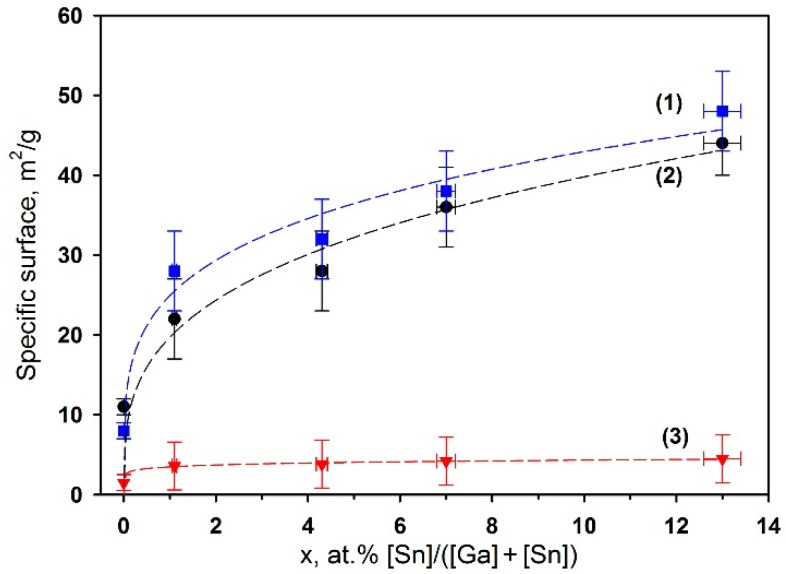
Specific surface of Ga_2_O_3_ samples annealed at 500 (**1**), 750 (**2**) and 1000 °C (**3**).

Such a change in the morphology and porous structure apparently determines the change in the specific surface area depending on the composition and annealing temperature of Ga_2_O_3_(Sn) (Figure 4). Thus, samples of the same composition annealed at 500 and 750 °C are characterized by a specific surface area that is the same within the experimental error. An increase in the annealing temperature up to 1000 °C leads to a dramatic decrease in the specific surface area. For all annealing temperatures, an increase in the surface area is observed with an increase in the tin concentration in Ga_2_O_3_(Sn).

### 3.4. XPS Study

**Figure 5:**
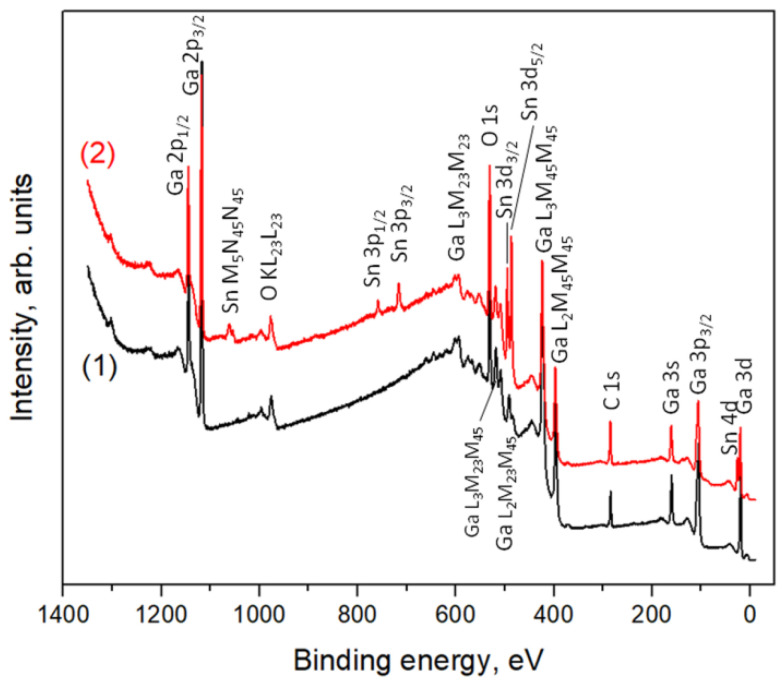
Survey XPS spectra of Ga_2_O_3_(Sn) annealed at 750 °C: (**1**) *x* = 0; (**2**) *x* = 13 at.% Sn. The main photoelectron and Auger lines of gallium and oxygen are identified.

Figure 5 shows the survey XPS spectrum (line (1)), which provides information on the surface composition of Ga_2_O_3_ sample obtained by annealing at 750 °C. The survey spectrum of a gallium oxide sample contains all the photoelectron and Auger lines of gallium and oxygen available for a given excitation radiation energy. There is also carbon on the sample surface, which is a typical surface contamination.

**Figure 6:**
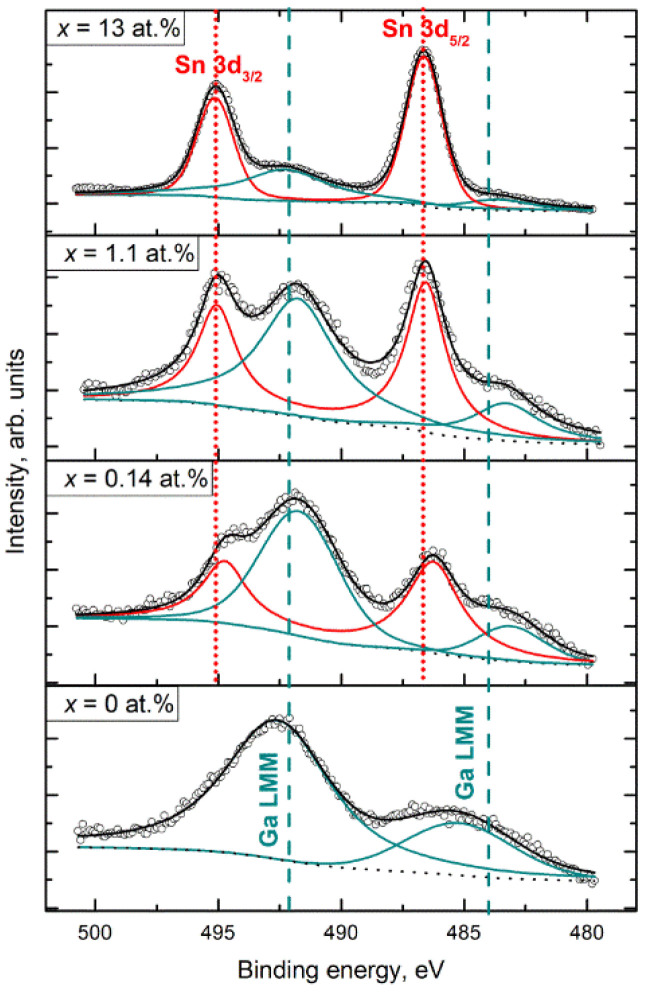
Sn3d XP spectra of the Ga_2_O_3_(Sn) samples annealed at 1000 °C.

The survey spectrum of Ga_2_O_3_(Sn) sample also shows characteristic photoelectron and Auger lines of tin along with the lines of gallium, oxygen, and carbon (Figure 5, line (2)). The XPS spectra contains a series of Auger lines of gallium in the range of binding energies from 370 to 670 eV. The presence of numerous gallium Auger lines in this region makes it difficult to analyze the spectra for the O1s and Sn3d regions (Figure 6) because of the peaks overlapping. The photoemission spectra of the Sn3d level are represented by a doublet Sn 3d_3/2_ (binding energy 495.1 eV) and Sn 3d_5/2_ (binding energy 486.7 eV) with spin-orbit splitting of 8.4 eV.

The O1s spectra split into three components located at 530.6–530.8 (O_L_), 531.2–531.4 (O_V_), and 532.4–532.6 (O_S_) eV (Figure S6, Supplementary materials). The O_L_ component can be ascribed to lattice oxygen in Ga_2_O_3_ and SnO_2_ oxides, while the O_V_ peak is associated with O^2−^ ions in the oxygen vacancies within Ga_2_O_3_ matrix (V_O_-like bonding [19]). The O_S_ component is due to the surface oxygen-containing species. The deconvolution of O1s spectra into components is not always unambiguous; however, it can be concluded that the contribution of O_S_ components to the spectra of all samples is 10–15% (Figure S6, Supplementary materials). The maximum contribution (18%) of the O_S_ component in O1s spectrum was revealed in the case of Ga_2_O_3_(Sn) (x = 0.14 at.%) annealed at 750 °C.

Table 2 shows the quantitative cation composition of the samples, determined from the X-ray photoelectron spectra. In all cases, the determined surface tin content exceeds that defined by ICP-MS measurements that can be associated with two factors: (i) a high calculation error due to the superposition of XPS lines in the regions corresponding to the analytical lines of elements and (ii) the inhomogeneous composition along the depth of the sample. In the samples with high tin content SnO_2_ phase can be segregated on the surface of Ga_2_O_3_(Sn) grains; accordingly, the tin concentration on the surface will be much higher than in the bulk of the sample.

The data presented in Table 2 also reflect the oxygen non-stoichiometry of the samples. All samples of undoped gallium oxide (annealed at different temperatures) are characterized by the oxygen deficiency in comparison with the stoichiometric composition [Ga]:[O] = 1:1.5. With an increase in the tin concentration, the oxygen content increases that can be explained by the formation of SnO_2_ phase.

### 3.5. FTIR Spectroscopy Study

**Figure 7:**
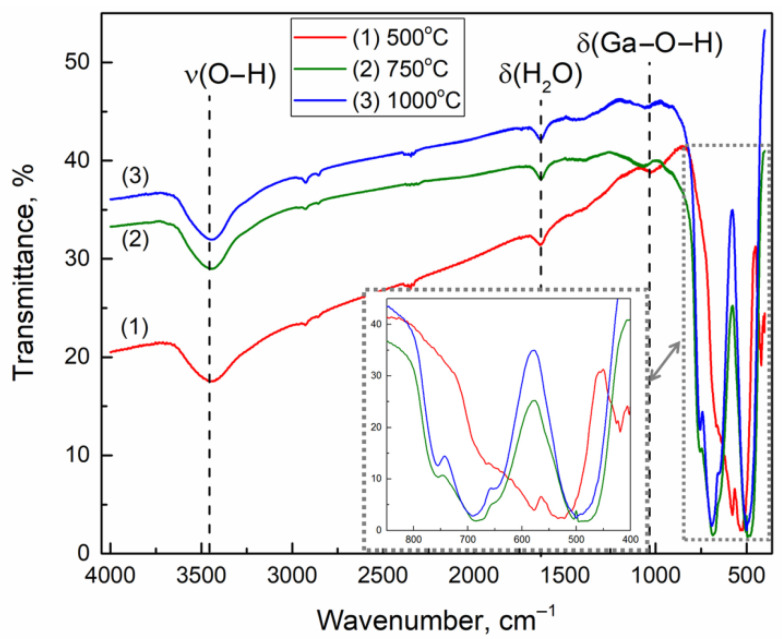
The FTIR spectra of Ga_2_O_3_ samples annealed at 500 (**1**), 750 (**2**) and 1000 °C (**3**). The inset shows the data in the range of 400–850 cm^−1^.

The FTIR absorption spectra of Ga_2_O_3_ samples annealed at different temperatures (Figure 7) indicate the presence of adsorbed water molecules (peak of deformation vibrations at 1640 cm^−1^), as well as OH groups (peak of stretching vibrations at 3400 cm^−1^ and bending vibrations of Ga–O–H at 1030 cm^−1^). The two complex peaks in the ranges of 450–530 cm^−1^ and 650–760 cm^−1^ for samples annealed at 750 and 1000 °C are typical for β-Ga_2_O_3_ lattice structure and represent the superposition of 4 A_u_ and 8 B_u_ broadened IR-active modes [20,21]. The IR spectrum of Ga_2_O_3_ sample annealed at 500 °C has multiple peaks in the ranges of 520–650 cm^−1^ and 401–430 cm^−1^ and is typical for α-Ga_2_O_3_ [21].

**Figure 8:**
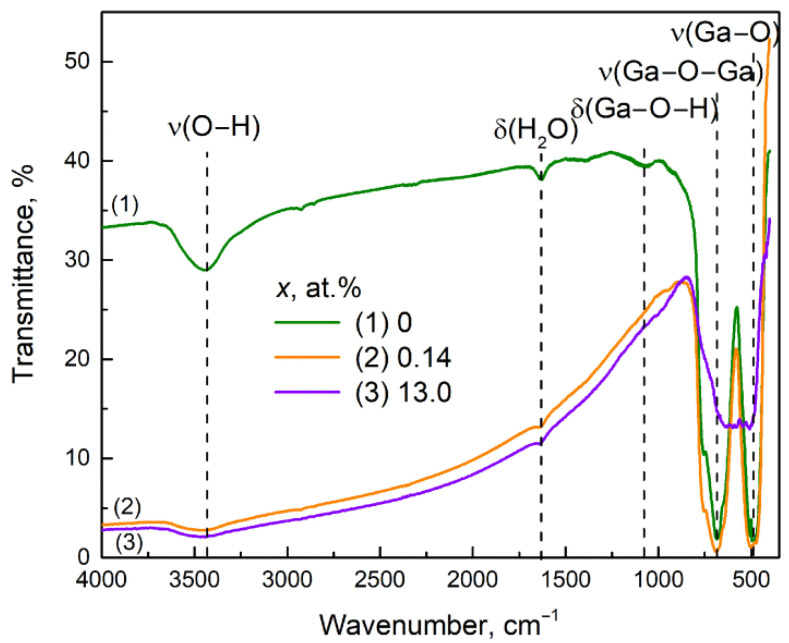
The FTIR spectra of Ga_2_O_3_(Sn) samples annealed at 750 °C.

The FTIR absorption spectra of Ga_2_O_3_(Sn) samples annealed at 750 °C (Figure 8) indicate that the introduction of 0.14 at.% Sn does not affect the peaks of the lattice Ga–O and Ga–O–Ga vibrations apparently due to the incorporation of Sn(IV) into the Ga_2_O_3_ crystal lattice. The increase in tin content up to 13 at.% leads to the decrease in intensity and spread out in width of observed peaks. This indicates the segregation of SnO_2_ on the surface of Ga_2_O_3_ crystallites and the contribution of stretching Sn–O vibrations (570 cm^−1^) to the absorption in this spectral region. Intense background absorption of doped samples may be due to the increased concentration of free electrons as compared to undoped Ga_2_O_3_. The substitution of Ga^3+^ cations by Sn^4+^ cations can be described with the following quasi-chemical reaction (the notations of point defects are given according to Kroeger-Wink):

(4) $$2{\mathrm{SnO}}_{2}\overset{Ga_{2}O_{3}}{\to }2{\mathrm{Sn}}_{Ga}^{∙}+3O_{O}^{\times }+2\bar{e}+\frac{1}{2}O_{2\left(gas\right)},$$

thus, the incorporation of Sn^4+^ into the Ga^3+^ position leads to the release of an electron. In the case of nonstoichiometric gallium oxide Ga_2_O_3-δ_ the doping with tin can be represented as a result of the interaction of Sn^4+^ cations with intrinsic defects in the gallium oxide structure, namely ionized oxygen vacancies and/or interstitial gallium atoms, for example:

(5) $$2{\mathrm{SnO}}_{2}+V_{O}^{∙}\to 2{\mathrm{Sn}}_{Ga}^{∙}+4O_{O}^{\times }+\bar{e}.$$

During these processes, the phase composition remains β-Ga_2_O_3_ as for undoped sample. The introduction of more than 0.23 at.% Sn leads to the formation of α-Ga_2_O_3_ phase as it was shown by XRD.

### 3.6. DRIFT Spectroscopy

#### 3.6.1. CO_2_ Adsorption

**Figure 9:**
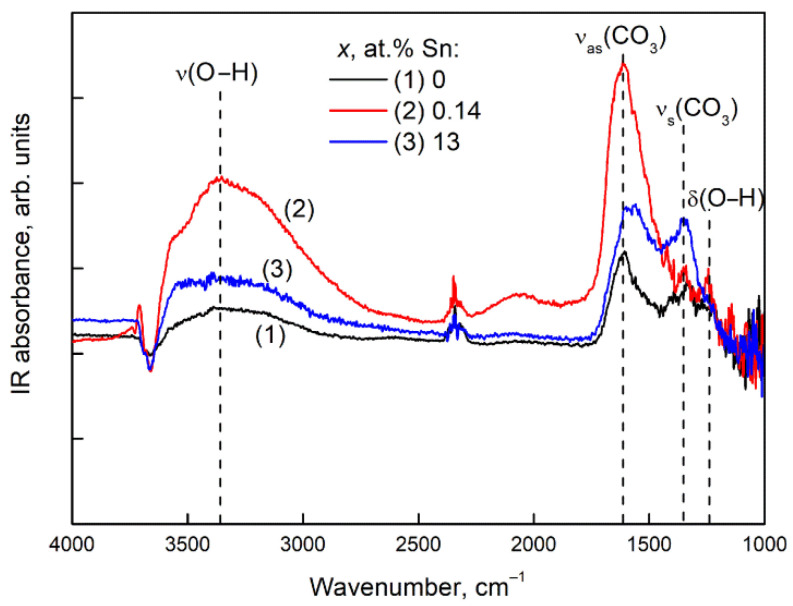
DRIFT spectra of Ga_2_O_3_(Sn) samples after exposure for 1 h in CO_2_ (500 ppm) in air at room temperature: (**1**) 0, (**2**) 0.14, (**3**) 13 at.% Sn.

Figure 9 shows the DRIFT spectra of Ga_2_O_3_(Sn) samples after 1 h exposure in CO_2_ (500 ppm) in air at room temperature.

The bands in the ranges of 1340–1330 cm^−1^ and 1660–1540 cm^−1^ are characteristic for carbonate and bicarbonate groups [22]. The carbonate species result from the adsorption of carbon dioxide across the M–O (M is a metal ion) bridge sites on the surface of oxide material. The C atom of CO_2_ molecule is bound to surface O atom; the O atom of CO_2_ is bound to a surface M atom. As a result, during the reaction

(6) $${\mathrm{CO}}_{2\left(gas\right)}+O_{\left(surface\right)}^{2-}={\mathrm{CO}}_{3\left(ads\right)}^{2-}$$

different types of surface carbonate species are formed: bridged carbonate (CO_3_^2−^ group bounds to two different metal cations), bidentate carbonate (CO_3_^2−^ group forms two bonds with one metal cation) or polydentate carbonate (CO_3_^2−^ group forms three bonds with three different metal cations). The formation of bicarbonate surface groups can be explained by the protonation of carbonate species by the hydroxyl OH-groups of the material surface [23]:

(7) $${\mathrm{CO}}_{3\left(ads\right)}^{2-}+{\mathrm{OH}}_{\left(surface\right)}^{-}={\mathrm{HCO}}_{3\left(ads\right)}^{-}+O_{\left(surface\right)}^{2-}.$$

In addition, the formation of bicarbonate species can be caused by the reaction of more basic surface hydroxyl groups with CO_2_ [24]:

(8) $${\mathrm{CO}}_{2\left(gas\right)}+{\mathrm{OH}}_{\left(surface\right)}^{-}={\mathrm{HCO}}_{3\left(ads\right)}^{-}.$$

The bicarbonate group may be mono- and bidentate coordinated to the metal oxide surface [24].

The increase in absorption in the region of O–H stretching vibrations can be associated with the concomitant adsorption of water in the presence of CO_2_. In the range of 3715 and 3612 cm^−1^, IR combination bands/overtones of the gas-phase CO_2_ is also observed [24]. With the introduction of tin in Ga_2_O_3_ structure the adsorption of CO_2_ increases most of all with the introduction of 0.14 at.% Sn. This fact may be explained by the increase in the concentration of basic surface centers, such as chemisorbed oxygen ions. Since Sn^4+^ cations act as electron donors (see Equation (4)), their presence can promote oxygen adsorption via reaction:

(9) $$\beta /2O_{2\left(gas\right)}+\alpha e^{-}↔O_{\beta \left(ads\right)}^{-\alpha },$$

so as to yield reactive surface oxygen ions $O_{\beta \left(ads\right)}^{-\alpha }$, which are necessary for CO_2_ adsorption Equation (6). The appearance of a weak peak at 2060 cm^−1^ may be due to the overtone of Ga–O–H bending vibrations. The band in the range of 2370–2310 cm^−1^ is attributed to the weak linearly or physically adsorbed CO_2_ on the surface of the materials [23].

With an increase in the tin content up to 13 at.% Sn, the peaks of the adsorbed carbonate ions decline due to a decrease in the basicity of the surface upon the substitution of Ga^3+^ for Sn^4+^. Nevertheless, the adsorption of CO_2_ on the surface of Ga_2_O_3_(Sn) is higher than on the surface of pure Ga_2_O_3_ annealed at the same temperature (750 °C) probably due to the larger specific surface area of Ga_2_O_3_(Sn) containing 13 at.% Sn.

#### 3.6.2. NH_3_ Adsorption

**Figure 10:**
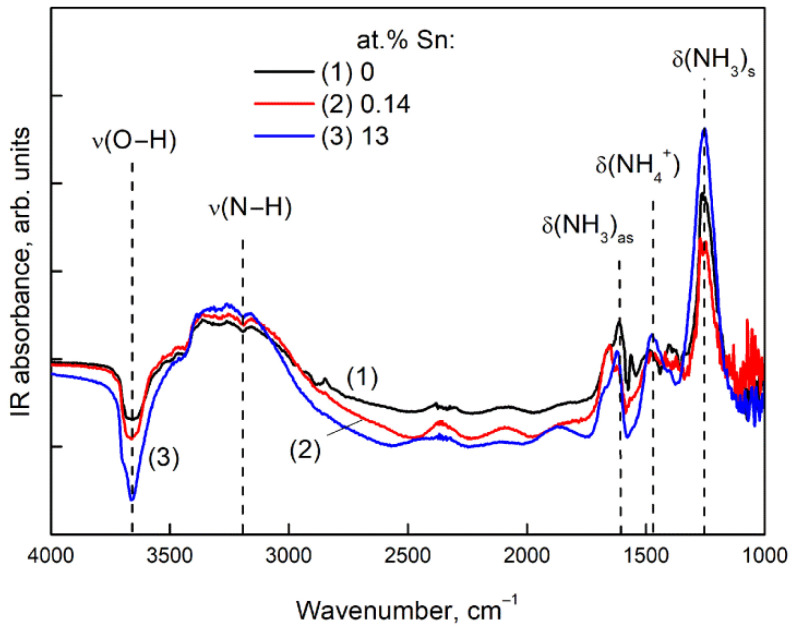
DRIFT spectra of Ga_2_O_3_(Sn) samples after exposure for 1 h in NH_3_ (200 ppm) in air at room temperature: (**1**) 0, (**2**) 0.14, (**3**) 13 at.% Sn.

The DRIFT spectra of Ga_2_O_3_(Sn) samples held for 1 h in the presence of NH_3_ (200 ppm) in air at room temperature are represented in Figure 10.

The appearance of peaks at 1620 cm^−1^ and 1250 cm^−1^ is due to asymmetric and symmetric bending vibrations of NH_3_ molecules adsorbed on surface Ga^3+^ cations [25]:

(10) $${\mathrm{NH}}_{3\left(gas\right)}+{\mathrm{Ga}}_{\left(surface\right)}^{3+}={\mathrm{Ga}}^{3+}-{\mathrm{NH}}_{3\left(ads\right)}.$$

The appearance of a peak at 1480 cm^−1^ is due to bending vibrations of NH_4_^+^ ions adsorbed on acidic surface OH-groups:

(11) $${\mathrm{NH}}_{3\left(gas\right)}+{\mathrm{OH}}_{\left(surface\right)}=O-{\mathrm{NH}}_{4\left(ads\right)}.$$

The Equation (11) is also associated with a decrease in absorption in the region of O–H stretching vibrations, as well as the appearance of an absorption band for N-H stretching vibrations (3200 cm^−1^). The intensities of the adsorbed NH_3_ molecules and NH_4_^+^ ions peaks remain almost unchanged for Ga_2_O_3_ samples annealed at different temperatures. However, the introduction of tin enhances the adsorption of ammonia as can be observed by an increase in the peaks of NH_3_ bending vibrations (1250 cm^−1^) and a decrease in the peak of O–H stretching vibrations (3600 cm^−1^). This is due to the higher Lewis acidity of Sn^4+^ cations than Ga^3+^ ones. This effect is more significant for samples with high tin concentration, apparently due to the segregation of SnO_2_ on the Ga_2_O_3_(Sn) surface. At a concentration of 0.14 at.%, Sn did not affect the intensity of the peaks of ammonia adsorption on the material surface, since Sn^4+^ cations were incorporated into the crystal lattice of Ga_2_O_3_, and the acidity of the surface did not change.

### 3.7. Sensor Properties

The sensor properties for the most samples obtained by the annealing at 500 °C cannot be measured because of high (more than 10^11^ Ohm) resistance. The sensor properties were studied for Ga_2_O_3_(Sn) samples annealed at 750 and 1000 °C.

#### 3.7.1. CO Gas Response

**Figure 11:**
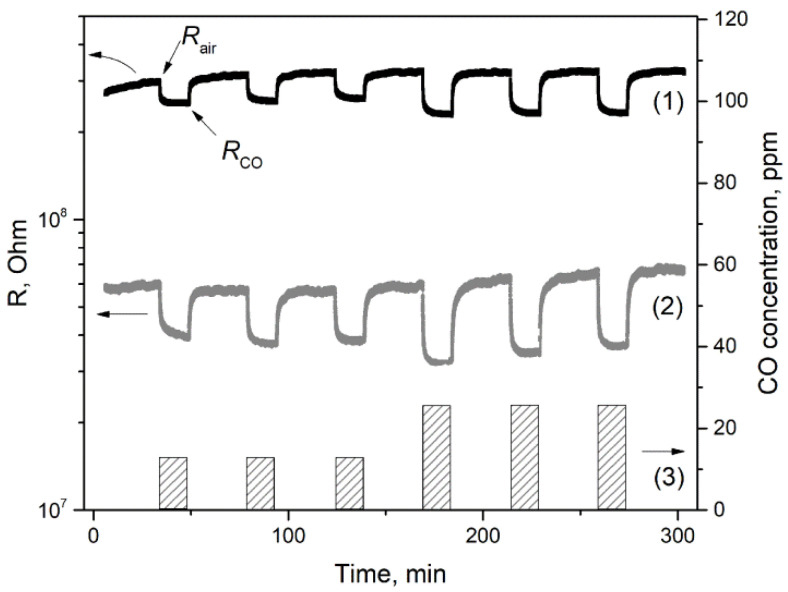
The resistance response of Ga_2_O_3_(0.14%Sn) annealed at 750 °C (**1**) and Ga_2_O_3_(13%Sn) annealed at 1000 °C (**2**) to 12.8 and 25.6 ppm CO pulses (**3**). Operating temperature is 500 °C.

In the presence of carbon monoxide, the electrical resistance of all samples decreases (Figure 11). This is consistent with the fact that CO reacts with oxygen chemisorbed on the surface of gallium oxide in accordance with the following reaction [3,26]:

(12) $${\beta \mathrm{CO}}_{\left(gas\right)}+O_{\beta \left(ads\right)}^{-\alpha }\to {\beta \mathrm{CO}}_{2\left(gas\right)}+\alpha e^{-},$$

where CO_(*gas*)_ is a carbon monoxide molecule in the gas phase, $O_{\beta \left(ads\right)}^{-\alpha }\;$ is a particle of chemisorbed oxygen on the surface of a semiconductor material, *ē* is an electron that is injected into the conduction band as a result of the reaction, CO_2(*gas*)_—molecule of reaction product CO_2_ desorbed from the surface of the material into the gas phase. It should be noted that the resistance of the samples returns to its original value in air atmosphere.

**Figure 12:**
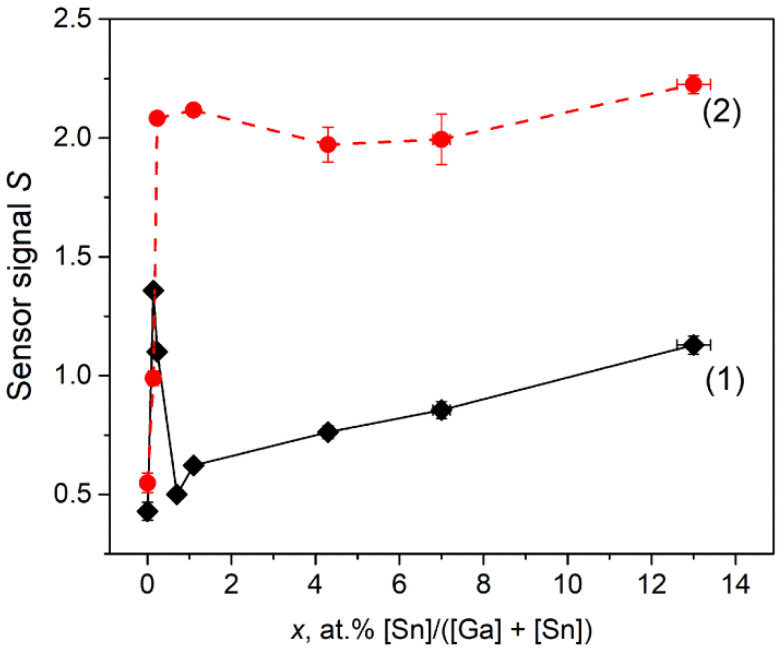
Sensor signal *S* to 256 ppm CO as a function of Sn content *x* for samples obtained by annealing at (**1**) 750 and (**2**) 1000 °C. The measuring temperature is 500 °C.

The dependences of Ga_2_O_3_(Sn) sensor signal on the tin content have non-monotonous character for both annealing temperatures of the samples (Figure 12). The introduction of small tin concentrations (0.14 at.% Sn for annealing temperature of 750 °C and 0.23 at.% Sn for annealing temperature of 1000 °C) leads to the sharp increase in sensor signal values. It can be assumed that such an increase in the sensor response is due to an increase in the concentration of chemisorbed oxygen on the surface of Ga_2_O_3_(Sn), which promotes the Equation (12).

With a further increase in the tin content, the dependences behave differently. As for the annealing temperature of 750 °C, the decrease in sensor signal with tin content of 0.14 < *x* < 0.7 at.% is attributed to the formation of low conductive α-Ga_2_O_3_ phase with corundum structure, thus the concentration of free electrons, which are necessary for reactive surface oxygen ions formation (Equation (9)), decreases rapidly and the Equation (12) yield decreases. For samples containing 0.7 < *x* < 13 at.% Sn, the gradual increase in sensor signal with tin content is observed that can be explained by formation of more conductive (as compared to α- and β-Ga_2_O_3_) SnO_2_ phase segregations. The *n-n* heterojunction between the Ga_2_O_3_ (bandgap *E_g_* = 4.9 eV [2], electron affinity χ = 4.0 eV [27]) and SnO_2_ (*E_g_* = 3.6 eV [26], electron affinity χ = 4.9 eV [28]) can also affect gas sensors properties of Ga_2_O_3_(Sn) materials. However, the possibility of mutual doping (formation of Sn-doped Ga_2_O_3_, *n*-type doping, and Ga-doped SnO_2_, *p*-type doping) complicates the interpretation of this effect. For the samples annealed at 1000 °C, the sensor signal is practically unchanged for Sn content of 0.23 < *x* < 13 at.%. As the main phase for all samples annealed at 1000 °C is β-Ga_2_O_3_, the sensor signal behavior indicates that tin cations act as electron donors in β-Ga_2_O_3_ structure in concentrations less than 0.23 at.%. The further increase in tin content does not lead to the growth in free electron concentration.

**Figure 13:**
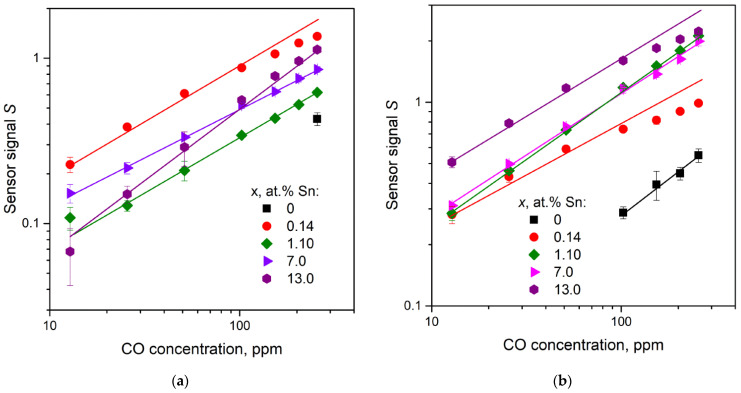
Sensor signal *S* as a function of CO concentration for Ga_2_O_3_(Sn) samples annealed at 750 (**a**) and 1000 °C (**b**). The measuring temperature is 500 °C.

The dependencies of sensor signal *S* on the CO concentration in air are represented in Figure 13. Tin-doped Ga_2_O_3_(Sn) samples allow to determine 12.8 ppm of CO while pure Ga_2_O_3_ samples were able to determine only high concentrations of carbon monoxide in air (256 and 102–256 ppm for Ga_2_O_3_ samples obtained at 750 and 1000 °C, respectively). The deviation from linearity of the plots at high CO concentrations may be due to incomplete desorption of CO_2_ or formation of surface polydentate carbonate species, which could occupy some adsorption sites and lead to a decrease in the sensor response. Carbon dioxide acts as Lewis acid and accepts electrons from the semiconductor material surface, and the growth of free electron concentration with Sn content leads to the increase in adsorbed CO_2_ molecules. It was shown that as compared to most forms of carbonate groups the polydentate carbonate groups [24] and bicarbonate species [27] are more stable and were observed on Ga_2_O_3_ surface at 450 °C and up to 477 °C, respectively. The adsorbed CO_2_ or polydentate carbonate species block the surface sites available for CO adsorption on Ga_2_O_3_(Sn).

#### 3.7.2. NH_3_ Gas Response

**Figure 14:**
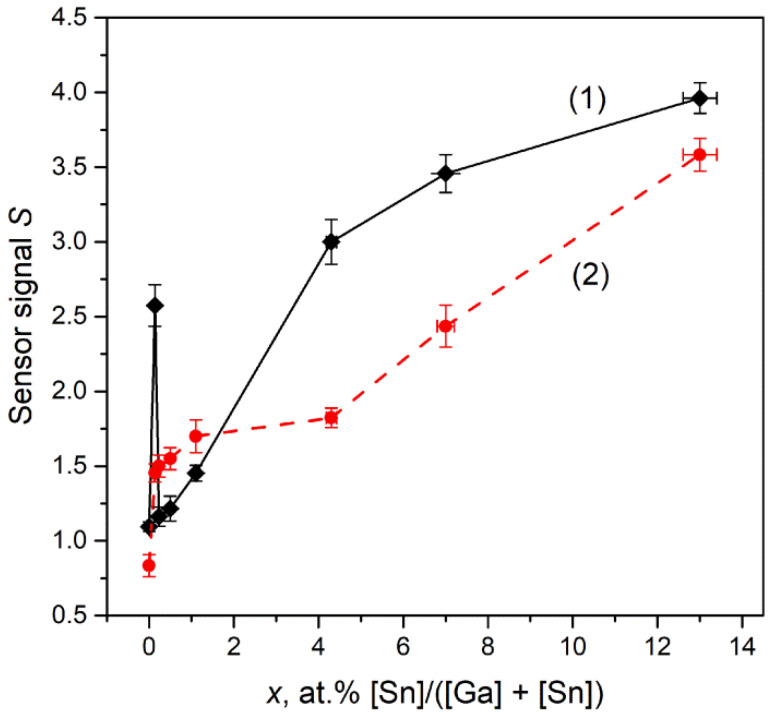
Sensor signal *S* to 155 ppm NH_3_ as a function of Sn content *x* for samples obtained by annealing at (**1**) 750 and (**2**) 1000 °C. The measuring temperature is 500 °C.

In the presence of ammonia, the electrical resistance of all samples also decreases: NH_3_ reacts with oxygen chemisorbed on the surface of semiconductor material in accordance with the following reaction [3,26]:

(13) $$2{\beta \mathrm{NH}}_{3\left(gas\right)}+3O_{\beta \left(ads\right)}^{-\alpha }\to {\beta N}_{2\left(gas\right)}+3{\beta H}_{2}O_{\left(gas\right)}+3\alpha e^{-},$$

where NH_3(*gas*)_ is an ammonia molecule in the gas phase, $O_{\beta \left(ads\right)}^{-\alpha }\;$ is a particle of chemisorbed oxygen on the surface of a semiconductor material, *ē* is an electron that is injected into the conduction band as a result of the reaction, N_2(*gas*)_ and H_2_O_(*gas*)_–molecules of reaction products desorbed from the surface of the material into the gas phase. The dependences of Ga_2_O_3_(Sn) sensor signal to ammonia on the tin content are represented in Figure 14. As it was obtained for CO detection, the introduction of small Sn concentrations (*x* = 0.14 at.%) leads to the sharp increase in sensor response that can be explained by the increase in chemisorbed oxygen ions concentration by Equation (9). The decrease in sensor signal for samples annealed at 750 °C with tin content of 0.14 < *x* < 0.23 at.% may be attributed to the formation of low conductive α-Ga_2_O_3_ phase as discussed above. The increase in sensor response with tin content for samples containing 0.23 < *x* < 13 at.% Sn can be explained by more acidic surface properties of the material. As it was shown in Section 3.6.2, introduction of tin enhances the adsorption of ammonia because of the higher Lewis acidity of Sn^4+^ cations than Ga^3+^ ones.

**Figure 15:**
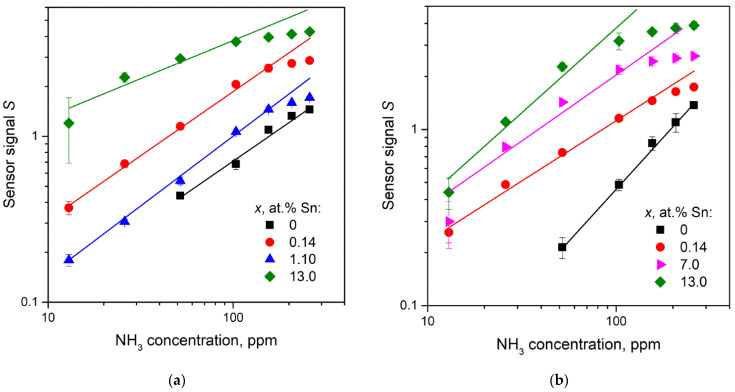
Sensor signal *S* as a function of NH_3_ concentration for Ga_2_O_3_(Sn) samples annealed at 750 (**a**) and 1000 °C (**b**). The measuring temperature is 500 °C.

Figure 15 illustrates a bilogarithmic plot of the sensor signal *S*–ammonia concentration dependencies for some Ga_2_O_3_(Sn) samples.

The deviation from linearity of the plots at high NH_3_ concentrations may be due to a competitive adsorption of NH_3_ and H_2_O on Ga_2_O_3_(Sn). The interaction of two ammonia molecules with chemisorbed oxygen leads to the formation of three water molecules according to the Equation (13). The interaction of water molecules with semiconductor material surface is more favorable by the mechanism of dissociative adsorption. The adsorption of H_2_O molecule requires one surface metal cation (Sn or Ga) and one surface oxygen. During this process, two constituents form: a hydrogen atom H_(*ads*)_, which binds to one surface oxygen atom, and surface hydroxyl group OH_(*ads*)_, binds to a metal surface atom. Thus, the adsorption of H_2_O will decrease the availability of surface-active sites for NH_3_ adsorption and reaction. Although the water adsorption on Ga_2_O_3_ and SnO_2_ surfaces is a thermodynamically favorable exothermic process, the values of the adsorption energy are −0.62 eV [29] or −60 kJ/mol for Ga_2_O_3_ and −150 kJ/mol for SnO_2_ [30]. Thus, the introduction of tin into the Ga_2_O_3_(Sn) leads to an increase in the adsorption of water and a decrease in the concentration of active centers on the surface, which are necessary for Equation (13).

## 4. Conclusions

Ga_2_O_3_ and Ga_2_O_3_(Sn) samples with tin content of 0–13 at.% were prepared by aqueous co-precipitation method with further annealing at 500, 750 and 1000 °C. The introduction of tin leads to a decrease in the average crystallite size and to the increase in the specific surface area. The phase transition of metastable α-Ga_2_O_3_ into thermodynamically stable β-Ga_2_O_3_ occurs at 740 °C for pure Ga_2_O_3_ sample; presence of tin shifts the temperature of β-Ga_2_O_3_ formation for more than 750 °C, also other metastable Ga_2_O_3_ phases are observed. Annealing at 1000 °C leads to the formation of only β-Ga_2_O_3_ and SnO_2_ phases. The minimum concentration at which the SnO_2_ phase is observed by XRD is 13 at.% for annealing temperature 500 °C, 7.0 at.% for 750 °C and 0.7 at.% for 1000 °C. The sensor responses of all Ga_2_O_3_(Sn) samples to CO and NH_3_ are typical for a n-type semiconductor and have non-monotonous character.

The study of CO_2_ probe molecule adsorption, gas responses to CO and NH_3_ indicate that small amounts of tin (0 < *x* < 0.14 at.%) are incorporated in Ga_2_O_3_ structure. Sn^4+^ cations act as electron donors and promote oxygen adsorption and formation of reactive chemisorbed oxygen ions $O_{\beta \left(ads\right)}^{-\alpha }$ (basic surface centers). These chemisorbed oxygen ions are responsible for oxidation of CO and NH_3_ on Ga_2_O_3_(Sn) surface at 500 °C and, respectively, the sharp increase in sensor signal. The further increase in tin content does not lead to the growth in free electron concentration.

The change in the sensor signal with Sn content depends on the annealing temperature of the samples. As for annealing temperature of 750 °C, the decrease in sensor signal with tin content of 0.14 < *x* < 0.7 at.% is caused by the formation of low conductive α-Ga_2_O_3_ phase. This decrease in sensor response is not observed for samples annealed at 1000 °C because they consist of β-Ga_2_O_3_ phase.

XPS and FTIR spectroscopy data indicate that tin dioxide phase can be segregated on the surface of Ga_2_O_3_(Sn) grains at high Sn contents; accordingly, the tin concentration on the surface is much higher than in the bulk of the sample. The CO probe molecule study shows a decrease in the basicity of the surface upon the substitution of Sn(IV) for Ga(III) for high tin concentrations (13 at.%). The introduction of tin enhances the adsorption of ammonia due to the higher Lewis acidity of Sn^4+^ cations than Ga^3+^ ones. The increase in sensor signal with tin content for samples containing 0.23 < *x* < 13 at.% Sn can be explained by more acidic surface properties of the material. The increase in sensor signal for samples containing 0.7 < *x* < 13 at.% Sn can be explained by formation of more conductive (as compared to α- and β-Ga_2_O_3_) SnO_2_ phase segregations.

Thus, tin additive in gallium(III) oxide influence on the material properties in a complex way: at low tin concentrations the generated donor centers and, respectively, the increase in oxygen chemisorption are more significant; at high concentrations of Sn, tin dioxide segregation on the surface of Ga_2_O_3_(Sn) grains and a change in the phase composition (for the samples annealed at 750 °C) become main determinants of surface and gas sensor properties.

## Figures and Tables

**Table 1:** Phase composition of Ga_2_O_3_(Sn) samples.

Sn Content *x*, at.% [Sn]/([Ga] + [Sn]) (ICP-MS)	Annealing Temperature, °C		
	500	750	1000
0 ≤ *x* ≤ 0.23	α-Ga_2_O_3_	β-Ga_2_O_3_	β-Ga_2_O_3_
0.50 ± 0.04	α-Ga_2_O_3_	β-Ga_2_O_3_, α-Ga_2_O_3_	β-Ga_2_O_3_
0.70 ± 0.02	α-Ga_2_O_3_	β-Ga_2_O_3_, α-Ga_2_O_3_, ε-Ga_2_O_3_	β-Ga_2_O_3_, SnO_2_
1.10 ± 0.05	α-Ga_2_O_3_	α-Ga_2_O_3_, β-Ga_2_O_3_, ε-Ga_2_O_3_	β-Ga_2_O_3_, SnO_2_
4.30 ± 0.12	α-Ga_2_O_3_, ε-Ga_2_O_3_	α-Ga_2_O_3_, β-Ga_2_O_3_, ε-Ga_2_O_3_	β-Ga_2_O_3_, SnO_2_
7.0 ± 0.2	α-Ga_2_O_3_, ε-Ga_2_O_3_	α-Ga_2_O_3_, β-Ga_2_O_3_, ε-Ga_2_O_3_, SnO_2_	β-Ga_2_O_3_, SnO_2_
13.0 ± 0.4	α-Ga_2_O_3_, ε-Ga_2_O_3_, SnO_2_	α-Ga_2_O_3_, ε-Ga_2_O_3_, SnO_2_	β-Ga_2_O_3_, SnO_2_

**Table 2:** XPS characterization of Ga_2_O_3_(Sn) samples.

Sn Content *x*, at.% [Sn]/([Ga] + [Sn]) (ICP-MS)	Annealing Temperature, °C					
	500		750		1000	
	Sn Content, at.% (XPS)	[Ga]:[O] Ratio	Sn Content, at.% (XPS)	[Ga]:[O] Ratio	Sn Content, at.% (XPS)	[Ga]:[O] Ratio
0	0	1:1.02	0	1:1.11	0	1:1.15
0.14 ± 0.02			1.4 ± 0.2	1:1.24	3.1 ± 0.3	1:1.33
1.10 ± 0.05	3.6 ± 0.4	1:1.30	3.2 ± 0.3	1:1.24	4.5 ± 0.5	1:1.28
13.0 ± 0.4	20 ± 2	1:1.62	17 ± 2	1:1.54	15 ± 2	1:1.51

## Data Availability

The data presented in this study are available on request from the corresponding author. The data are not publicly available due to privacy reason.

## Supplementary Materials

The following are available online at https://www.mdpi.com/article/10.3390/nano11112938/s1, Table S1: Starting materials for the synthesis of Ga_2_O_3_(Sn) powders, Figure S1: The XRD data of powders obtained by the interaction of gallium and tin salts solution with ammonia: (1) 0, (2) 1.1, (3) 4.3, (4) 7.0, (5) 13 at.% Sn. Gray vertical lines indicate ICDD GaOOH (6–180) data, Figure S2: MS data of gaseous products formed by the decomposition of precursor containing (a) 0 and (b) 13 at.% Sn, Figure S3: XRD patterns of Ga_2_O_3_(Sn), annealed at 500 °C: (1)–0, (2)–1.10, (3)–4.3, (4)–7.0, (5)–13 at.% Sn. Vertical gray bars indicate positions and relative intensities of α-Ga_2_O_3_ (ICDD card [43–1013]). The peaks of other phases (probable ε-Ga_2_O_3_) are marked by (*), Figure S4: XRD patterns of Ga_2_O_3_(Sn), annealed at 750 °C, Figure S5: XRD patterns of Ga_2_O_3_(Sn), annealed at 1000 °C, Figure S6: O1s spectrum of Ga_2_O_3_ (x = 0) annealed at 750 °C and fractions of different spectral components for Ga_2_O_3_(Sn), annealed at different temperatures.
