# Supplementary material for: Ga2O3(Sn) Oxides for High-Temperature Gas Sensors
Source: Nanomaterials (Basel). 2021 Nov 2;11(11):2938. doi: 10.3390/nano11112938 (PMC8624813; doi:10.3390/nano11112938)
Supplement: Supplementary file 1 [file nanomaterials-11-02938-s001.zip › nanomaterials-1427124-supplementary.pdf]

Supplementary Information

Ga_2O_3(Sn) Oxides for High-Temperature Gas Sensors

Nataliya Vorobyeva [1], Marina Rumyantseva [1,*], Vadim Platonov [1], Darya Filatova [1], Artem Chizhov [1], Artem Marikutsa [1], Ivan Bozhev [2,3] and Alexander Gaskov [1]

[1] Chemistry Department, Moscow State University, 119991 Moscow, Russia; natali.vorobyeva@gmail.com (N.V.); agnes1992@yandex.ru (V.P.); gak1.analyt@gmail.com (D.F.); chizhov@inorg.chem.msu.ru (A.C.); artem.marikutsa@gmail.com (A.M.); gaskov@inorg.chem.msu.ru (A.G.)
[2] Quantum Technology Center, Moscow State University, 119991 Moscow, Russia; bozhjev.ivan@physics.msu.ru (I.B.)
[3] Faculty of Physics, Moscow State University, 119991 Moscow, Russia
* Correspondence: roum@inorg.chem.msu.ru (M.R.); Tel.: +7-495-939-5471

The synthesis of powders based on gallium (III) oxide was carried out by the method of coprecipitation from aqueous solutions of gallium (III) nitrate and tin (IV) chloride using an ammonia solution as a precipitant.

The starting materials used were:
• 8-aqueous gallium(III) nitrate $Ga(NO_3)_3{\cdot}8H_2O$ (analytical grade),
• 5-aqueous tin(IV) chloride $SnCl_4{\cdot}5H_2O$ (analytical grade),
• 25% ammonia solution (for analysis),
• distilled water.

Weighed portions of the starting materials are presented in Table S1. The content of the doping additive (tin) is expressed in the atomic percent (the ratio of the number of tin cations to the total number of cations): [Sn]/([Ga]+[Sn])·100%. For samples with low tin content (0.1 – 0.8 at.% Sn), a tin(IV) chloride solution was used as a tin source, for which a 0.1500 g sample of $SnCl_4{\cdot}5H_2O$ was dissolved in 100 ml of distilled water.

The calculated amounts of gallium(III) nitrate and tin(IV) tetrachloride were dissolved in distilled water to form the solution with total metal ions concentration of 0.2 M. The initial ammonia solution was diluted twice with distilled water to obtain a concentration of 12.5%. Then, with constant stirring with a magnetic stirrer, an ammonia solution (12.5%) was added dropwise until a slightly alkaline reaction of the medium (to reach pH = 8 - 9, about 10 ml of ammonia solution was necessary for each sample). The formation of a white precipitate was observed, then the viscosity of the solution increased and it turned into a gel. Separation of sediments by centrifugation was carried out after aging (kept for 30 minutes at room temperature). The precipitate was separated by centrifugation (3000 rpm, 5 min) followed by decantation, then dispersed in distilled water and again separated by centrifugation. Similarly, the precipitate was washed with distilled water 5 times, then dried at 50°C for 24 hours in an oven and ground in an agate mortar. Annealing was carried out in a muffle furnace in air at temperatures of 500, 750, and 1000°C, the annealing time was 24 hours. After annealing, all Ga_2O_3(Sn) powders were ground in an agate mortar.

Table S1. Starting materials for the synthesis of Ga_2O_3(Sn) powders.

Sn content, at.%	Mass of $Ga(NO_3)_3{\cdot}8H_2O$, g	Mass of $SnCl_4{\cdot}5H_2O$, g	Volume of $SnCl_4$ solution, ml
0	8.5562	-	-
0.1	8.5476	-	5
0.2	8.5389	-	10
0.4	8.5218	-	20
0.6	8.5049	-	30
1	8.4704	0.0750	-
3	8.2993	0.2250	-
5	8.1282	0.3750	-
10	7.7004	0.7499	-

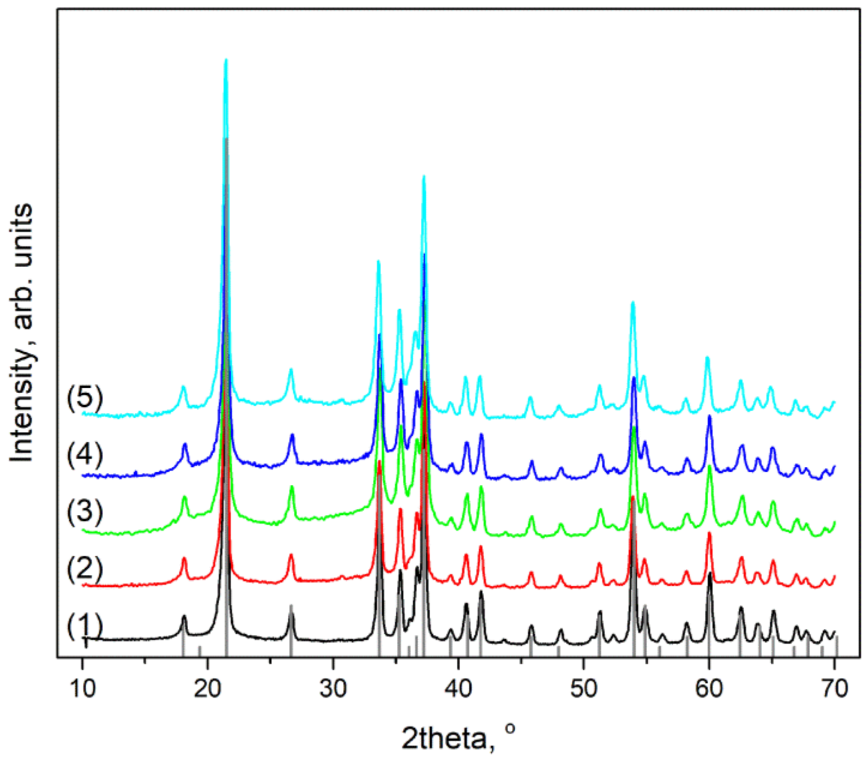

Figure S1. The XRD data of powders obtained by the interaction of gallium and tin salts solution with ammonia: (1) 0, (2) 1.1, (3) 4.3, (4) 7.0, (5) 13 at.% Sn. Gray vertical lines indicate ICDD GaOOH [6-180] data.

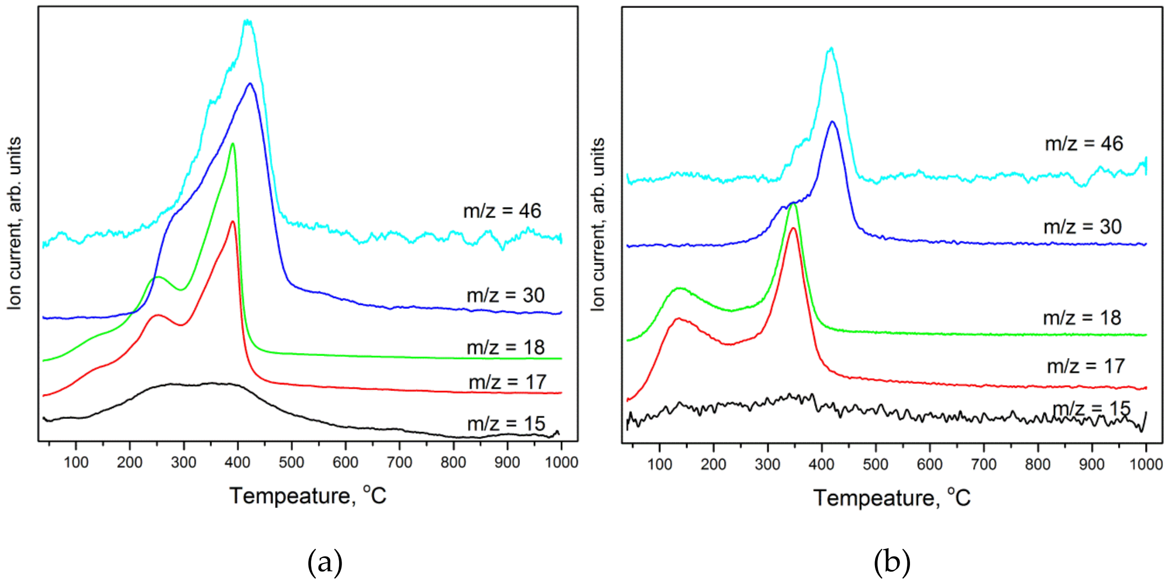

Figure S2. MS data of gaseous products formed by the decomposition of precursor containing (a) 0 and (b) 13 at.% Sn.

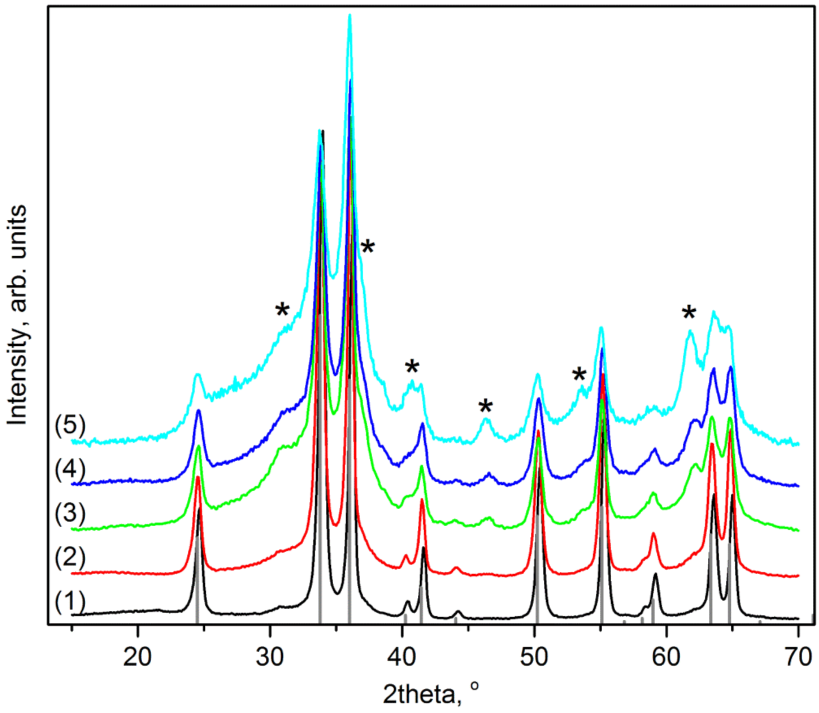

Figure S3. XRD patterns of Ga_2O_3(Sn), annealed at 500°C: (1) – 0, (2) – 1.10, (3) – 4.3, (4) – 7.0, (5) – 13 at.% Sn. Vertical gray bars indicate positions and relative intensities of α-Ga_2O_3 (ICDD card [43-1013]). The peaks of other phases (probable ε-Ga_2O_3) are marked by (*).

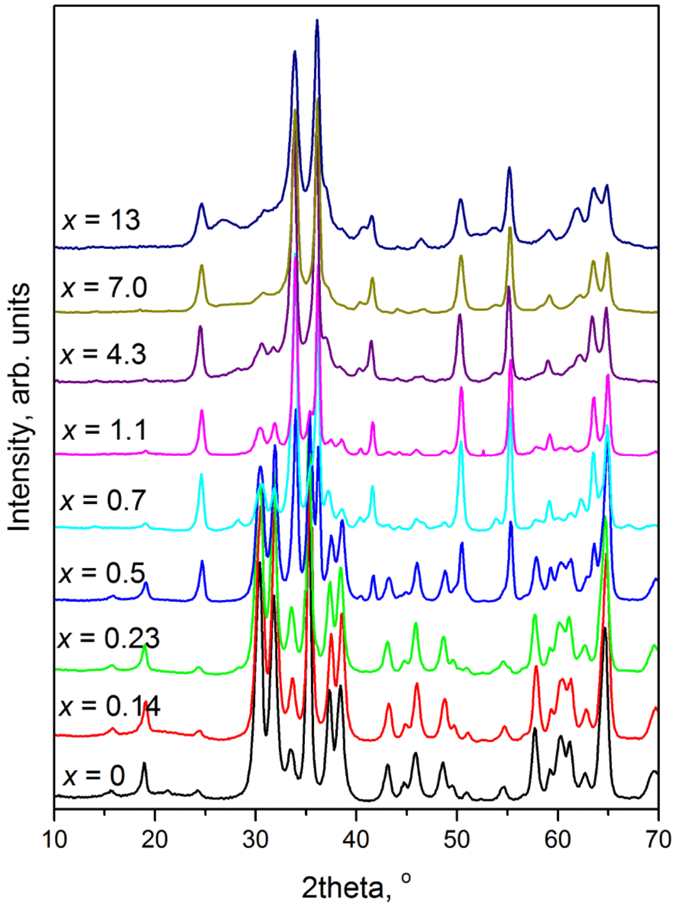

Figure S4. XRD patterns of Ga_2O_3(Sn), annealed at 750°C.

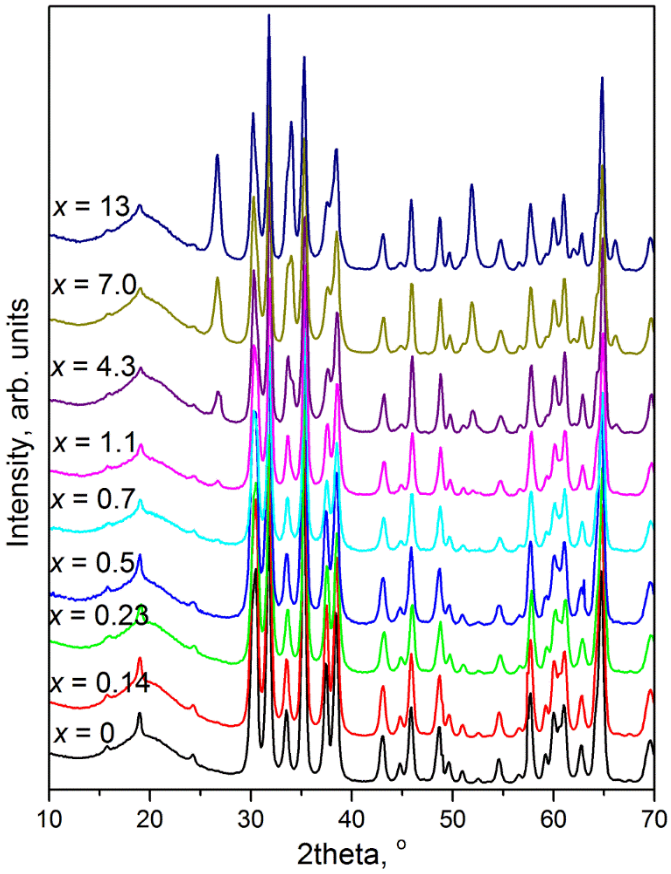

Figure S5. XRD patterns of Ga_2O_3(Sn), annealed at 1000°C.

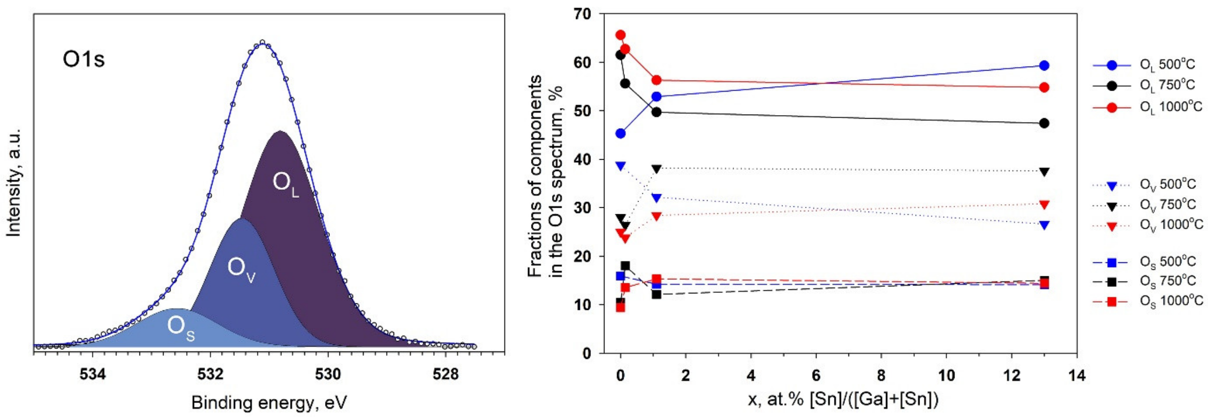

Figure S6. O1s spectrum of Ga_2O_3 (x = 0) annealed at 750°C and fractions of different spectral components for Ga_2O_3(Sn), annealed at different temperatures.
